# Optimization of the Microstructure and Mechanical Properties of a TC4 Alloy Joint Brazed with a Zr-Based Filler Containing a Co Element

**DOI:** 10.3390/ma17194861

**Published:** 2024-10-02

**Authors:** Zhan Sun, Deshui Yu, Lixia Zhang, Mingjia Sun, Boyu Zhang, Weimin Long, Sujuan Zhong

**Affiliations:** 1 State Key Laboratory of Precision Welding & Joining of Materials and Structures, Harbin 150001, China; hitsunzhan@outlook.com (Z.S.); yds960221@163.com (D.Y.); sunmingjia@163.com (M.S.); 524201736@163.com (B.Z.); 2 State Key Laboratory of Advanced Brazing Filler Metals & Technology, Zhengzhou Research Institute of Mechanical Engineering Co., Ltd., Zhengzhou 450001, China; longweimin@camsouth.com.cn (W.L.); zhongsujuan@126.com (S.Z.)

**Keywords:** TC4, erosion, vacuum brazing, Zr-based brazed filler, β-Ti phase

## Abstract

Herein, we fabricated a low-melting-point Zr-16Ti-6Cu-8Ni-6Co eutectic filler based on a Zr-Ti-Cu-Ni filler to achieve effective joining of a Ti6Al4V (TC4) titanium alloy. The temperature at which the brittle intermetallic compound (IMC) layer in the seam completely disappeared was reduced from 920 °C to 900 °C, which broadened the temperature range of the Zr-based filler, brazing the TC4 without a brittle IMC layer. The shear strength of the Zr-16Ti-6Cu-8Ni-6Co brazed joint increased by 113% more than that of the Zr-16Ti-9Cu-11Ni brazed joint at 900 °C. The proportion of β-Ti in the seam of the Zr-16Ti-6Cu-8Ni-6Co brazed joint increased by 21.31% compared with that of the Zr-16Ti-9Cu-11Ni brazed joint. The nano-indentation results show that the elastic modulus of the β-Ti (143 GPa) in the interface is lower than that of the α-Ti (169 GPa) and (Ti,Zr)_2_(Ni,Cu,Co) (203 GPa). As a result, the β-Ti is subjected to a greater strain under the same stress state compared with the α-Ti and (Ti,Zr)_2_(Ni,Cu,Co), and the Zr-16Ti-6Cu-8Ni-6Co brazed joint can maintain a higher strength than the Zr-16Ti-9Cu-11Ni brazed joint under a middle–low erosion area of the TC4 base metal. This provides valuable insights into the use of high-strength, fatigue-resistant TC4 brazed joints in engineering applications.

## 1. Introduction

Titanium (Ti) alloys are widely used in the aerospace, medical and health fields due to their excellent properties such as high strength, light weight, good erosion resistance and high temperature resistance [1]. Among them, the Ti6Al4V (TC4), as a typical α-β dual-phase Ti alloy, is suitable for situations with high comprehensive performance requirements, and its annual output accounts for more than half of the total Ti alloys [2]. Therefore, a TC4 alloy is used to prepare plate fin heat exchangers, which are one of the core components of marine and aerospace high-performance power systems. A TC4 plate fin heat exchanger with stacking multi-layer units is difficult to form integrally and has to be joined together. Among the various joining methods, brazing is more suitable for plate fin joining because of its advantages of a uniform heat input, small deformation of the base metal after joining, easy control of the residual stress and precise temperature control [3,4,5,6]. However, there are always erosion problems between the filler and the TC4 base metal during the brazing process. Zhang et al. [7] found that the erosion will be stronger as the brazing temperature increases. The brazing temperature and the type of braze fillers are the key factors to avoid the erosion of Ti alloys [8,9].

An appropriate selection of braze filler is particularly critical to solve the erosion problem in TC4 brazing. Commonly used fillers include Ag-based, Al-based, Ti-based and Zr-based braze fillers during brazing of Ti alloys. However, the strength of a Ti alloy brazed joint with an Ag-based filler at a high temperature is low, which means that it is difficult to meet the requirements of engineering applications. An Al-based filler will increase the Ti-Al brittle intermetallic compounds (IMCs) of Ti alloy brazed joints and reduce the impact strength. A Ti-based filler has good wettability, high-temperature strength and stable microstructure and properties during brazing of a Ti alloy [10,11]. At present, Ti-Cu-Ni and Ti-Zr-Cu-Ni series fillers are commonly used for brazing Ti alloys [12,13]. However, a Ti-based filler can easily cause erosion of TC4 thin-walled structures when the brazing temperature is too high. At present, the melting point of a Ti-based filler is usually above 830 °C, and the actual brazing temperature is above 980 °C [14,15]. He et al. [16] used a Ti-15Cu-15Ni filler to braze TC4 at 1020 °C, and there was a residual Ti_2_(Cu, Ni) IMC layer in the joint when the brazed temperature was below 1020 °C. Zhong et al. [17] found that there is a serious erosion problem of brazing Ti alloys with a general Ti-based filler, which causes accuracy issues with the brazed components. Zr and Ti elements have similar atomic characteristics, and the Zr-based filler has ultra-high strength, excellent erosion resistance and a low melting point [18], which is suitable for weak erosion brazing of TC4 alloys. Lee achieved a reliable TC4 brazed joint using a Zr-11.0Ti-13.2Cu-9.8Ni-3.3Be filler with a melting temperature of ~725 °C [19]. So, developing a Zr-based braze filler with a low melting point is promising in terms of addressing the erosion issues by brazing thin-walled TC4 structures at relatively low temperatures.

Low-temperature brazing of thin-walled TC4 structures with Zr-based braze fillers is beneficial to reduce the erosion of the plate fins. However, due to the insufficient interdiffusion at low temperatures, Cu and Ni can hardly diffuse into TC4 substrates, and more Cu and Ni elements will stay at the central joint, resulting in a residual brittle IMC layer in the brazed seam. When Ganjeh used a Ti-27Zr-14Cu-13Ni filler for TC4/Ti-CP brazing, Cu-Ni- or Cu-Ni-Zr-enriched phases (segregation zones) were detected from the fracture of the brazed joints [20]. The formation of brittle Cu-Ni IMCs in the seam should be avoided as much as possible during the brazing process, since it may induce a stress concentration, and the materials are prone to cracking. The β-Ti can dissolve more Cu and Ni elements (17.0% and 10.3%, respectively) [21] than α-Ti (1.6% and 0.5%, respectively) [19], and it has better ductility than α-Ti, increasing the content of β-Ti in the interface, which has the potential to improve the strength of the joint. The Co, V, Fe, etc., elements have stable β phase abilities in Ti alloys [22]. The β-Ti eutectoid reaction temperature of Ti-Co (β-Ti → α-Ti + Ti_2_Co) is the lowest at 685 °C compared with the Ti-Cu (800 °C) and Ti-Ni (765 °C) couples [23]. Therefore, the addition of the Co element may reduce the β-Ti eutectoid reaction temperature and expand the β-Ti phase region in low-temperature brazing.

Herein, we design and fabricate new Zr-based fillers (Zr-16Ti-6Cu-8Ni-6Co) with low melting points to braze a TC4 alloy based on a Zr-16Ti-9Cu-11Ni filler [24] and achieve a reliable joining of the TC4 alloy with minor erosion. Both the microstructure and mechanical performance of the TC4 brazed joints with Zr-based fillers (with and without Co addition) were compared. The mechanisms through which Co worked on the microstructural optimization and strength enhancement of the joint were discussed.

## 2. Experimental Procedures

Cuboid-shaped specimens were cut from the TC4 plate by electrical discharge machining. Before brazing, the oxide skin on the surface of the metal specimen was polished with sandpaper, and the polished base metal was placed in an ethanol solution for ultrasonic cleaning for 10 min. It was made into two cuboids of different sizes, and a filler foil was placed between the two cuboids to form a brazed joint.

In this study, Zr-16Ti-9Cu-11Ni and Zr-16Ti-6Cu-8Ni-6Co amorphous fillers were fabricated. Firstly, the filler metal block was prepared by arc melting. Zr-based amorphous fillers were prepared using an arc melting furnace (SP-MSM20-8, Hefei Kejing Materials Technology Co., Ltd., Hefei, China) and single roll conveyor machine (HVSD-2, Shenyang Haozhiduo New Materials Equipment Technology Co., Ltd., Shenyang, China). Ar gas was introduced into the smelting process, and the arc between the tungsten electrode and the workpiece was burned. The heat generated melted the metal particles, and the button-like sample was obtained after the copper crucible cooled. The single-roll conveyor heated the metal particles through the induction coil, and the molten metal was sprayed onto the high-speed rotating copper roller to prepare a ribbon-shaped amorphous filler. The crystallization temperature, solidus temperature and liquidus temperature of the Zr-based amorphous foil were measured using a differential scanning calorimetry (DSC) thermal analyzer (STA449F3, NETZSCH, Selb, Germany).

**Figure 1:**
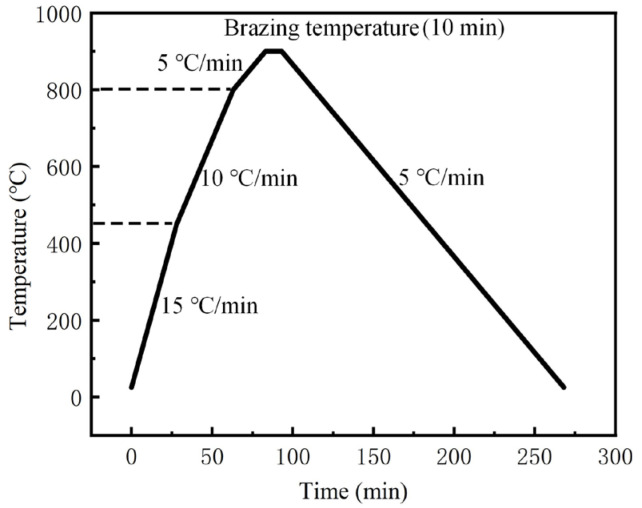
The heating and cooling curve of vacuum brazing.

The vacuum brazing equipment used in the test is independently developed by our research group, and the vacuum degree can reach 2 × 10^−4^ Pa when working. The maximum heating temperature can reach 1200 °C. The heating and cooling curve of vacuum brazing is shown in Figure 1. The whole brazing process is controlled by the input program: the temperature is increased from room temperature to 450 °C at a rate of 15 °C/min, then continues to rise to 800 °C at a rate of 10 °C/min, and finally increases at a rate of 5 °C/min until the final brazing temperature is reached, and the process is maintained at this temperature for a certain time. When the vacuum degree in the brazing equipment is lower than 5 × 10^−3^ MPa during brazing, the heating program is started. After the holding process, the temperature is reduced to 450 °C at a rate of 5 °C/min and then cooled with the brazing equipment.

The microstructure was characterized by an X-ray diffractometer (XRD, D8ADVANCE, Bruker Co., Ltd., Billerica, MA, USA) and field emission scanning electron microscope (SEM, Merlin Compact, Carl Zeiss AG, Oberkochen, Germany). The sample for transmission electronic microscopy (TEM, FEI Talos F200X, ThermoFisher Scientific, Hillsboro, OR, USA) observation was prepared by a focused ion beam (FIB, Gatan695, Gatan Inc., Pleasanton, CA, USA). The Pt strip was plated on the observation area, and the material around the Pt strip was etched with an FIB. Then, we continued to thin the sample with an FIB to about 500 nm. TEM analysis was performed at 200 kV. The local hardness degrees of different regions were measured by nano-indentation (G200). The maximum load is 26 mN and the loading rate is 0.01 mN·s^−1^. The elemental distribution in the interface microstructure was detected by an electron probe microanalysis (EPMA) at 20 kV. The shear strength of the brazed joint was measured using an electronic universal testing machine (AG-X Plus, Shimadzu Co., Ltd., Tokyo, Japan) (accuracy ± 0.5%), and the indentation speed in the shear test was set to 0.5 mm/min. To ensure the accuracy of the shear strength data, the shear strength values of the joint were the average of three samples at the same brazing temperature and test condition.

## 3. Results

### 3.1. Microstructure of the TC4 Brazed Joints

**Figure 2:**
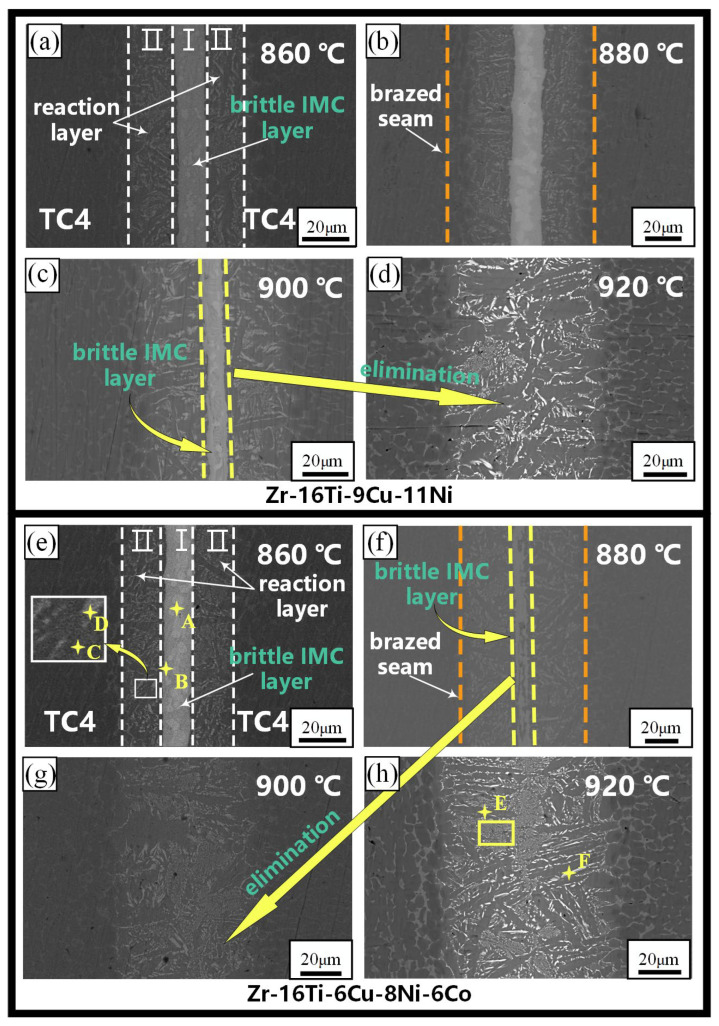
SEM images of brazed joints at different temperatures, Zr-16Ti-9Cu-11Ni brazed joint: (**a**) 860 °C, (**b**) 880 °C, (**c**) 900 °C, (**d**) 920 °C; Zr-16Ti-6Cu-8Ni-6Co brazed joint: (**e**) 860 °C, the interface microstructure includes (Ti,Zr)_(s.s)_ (marked by A), (Ti,Zr)_2_(Cu,Ni,Co) (marked by B), α-Ti (marked by C) and β-Ti (marked by D) (**f**) 880 °C, (**g**) 900 °C, (**h**) 920 °C, the interface microstructure includes α-Ti (marked by E) and β-Ti (marked by F). The region I and II is a brittle IMC layer and a reaction layer, respectively. With the increase of brazing temperature, the brazed seam (marked by orange dashed lines) is wider and the IMC layer (marked by yellow dashed lines) is thinner.

**Figure 3:**
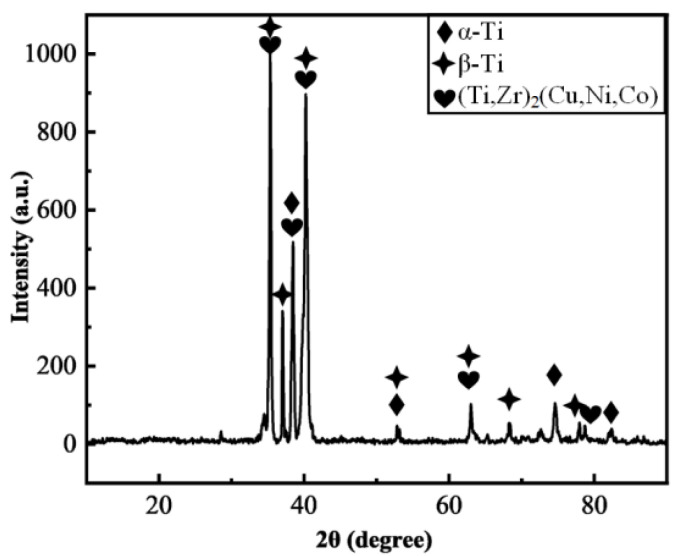
The XRD patterns of the brazed seam with a Zr-16Ti-6Cu-8Ni-6Co filler at 860 °C.

As shown in Figure 2, the Zr-16Ti-9Cu-11Ni (Figure 2a–d) and Zr-16Ti-6Cu-8Ni-6Co (Figure 2e–h) fillers are used to braze the TC4 as the temperature is increasing. The microstructure of the brazed joint is divided into three regions (Figure 2a,e), which are the brittle IMC layer (region I), reaction layer (region II) and TC4 base metal region. When the brazing temperature is lower than 900 °C, there is a brittle IMC layer in the center of the Zr-16Ti-9Cu-11Ni brazed seam (Figure 2c). As the temperature increases from 840 °C to 900 °C, the width of the brittle IMC layer decreases and disappears completely at 920 °C (Figure 2d). Nevertheless, the brittle IMC layer of the Zr-16Ti-6Cu-8Ni-6Co brazed joint disappears at 900 °C (Figure 2g). As shown in Table 1, the phases with different contrasts in the two typical interfaces with (860 °C, Figure 2e) and without a brittle IMC layer (920 °C, Figure 2h) were scanned by point EDX analysis. The proportion of Ti and Zr elements in the bright white phase A in region I of the two joints at 840 °C is more than 90%, while the contents of other elements are lower, and the Ti and Zr elements are infinitely miscible. Therefore, it is speculated that the bright white phase A is (Ti,Zr)_(s.s)_. The dark phase B is the main phase in the center of region I. Combined with the element content and thermodynamics reaction, it is speculated that the B phase is (Ti,Zr)_2_(Cu,Ni,Co) IMCs, which may be formed by residual fillers. The content of Ti in the dark phase C in region II is 80.71%, and the content of Cu and Ni is lower than its limit solubility in α-Ti (1.6% and 0.5%, respectively) [19]. Therefore, the C phase is α-Ti with a certain amount of Zr dissolved. The contents of Ti in the bright white phase D is 64.47%. The contents of Cu and Ni are higher than their limit solubility in α-Ti and lower than their limit solubility in β-Ti (17.0% and 10.3%, respectively) [21]. Cu and Ni are stable elements of β-Ti, so it is speculated that the D phase is β-Ti, in which a certain amount of Zr is dissolved. The element content of the dark phase E is close to that of the C phase, and the element content of the bright white phase F is close to that of the D phase. Combined with the element content, morphology and contrast, it is speculated that E is α-Ti, and the F phase is β-Ti. The Zr-16Ti-6Cu-8Ni-6Co brazed joint at 860 °C includes (Ti,Zr)_2_(Cu,Ni,Co), α-Ti and β-Ti phases (Figure 3). When the brazing temperature is higher than the melting temperature (900 °C) of the brittle IMC layer, the interface microstructure of the Zr-16Ti-6Cu-8Ni-6Co brazed joint is composed of α-Ti and β-Ti. The bright white phase is β-Ti, and the dark phase is α-Ti. The tiny (Ti,Zr)_2_(Cu,Ni,Co) phase in the eutectoid microstructure is not visible at a low magnification. In the Zr-16Ti-6Cu-8Ni-6Co brazed joint, when the brazing temperature is higher than 900 °C, the interface microstructure is a dense α-Ti+β-Ti eutectoid microstructure. As the temperature continues to increase, a coarse strip of α-Ti appears throughout the seam. Comparing the brazed seam microstructure of the brazed joints of the two kinds of fillers, it can be seen that the temperature of the brittle IMC layer disappears in the seam center following a reduction from 920 °C to 900 °C after adding a Co element. Therefore, a reliable TC4 joint can be achieved at a lower temperature by the Zr-based filler with Co added.

**Figure 4:**
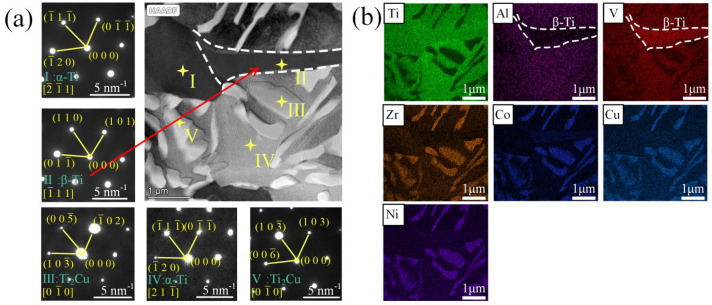
TEM images of microstructures in Figure 2h: (**a**) High-angle annular dark-field imaging, (HAADF) and selected area electron diffraction (SAED), the interface microstructure includes α-Ti (marked by I), β-Ti (marked by II), (Ti,Zr)_2_(Cu,Ni,Co) (marked by III), α-Ti (marked by and IV), and (Ti,Zr)_2_(Cu,Ni,Co) (marked by V); (**b**) elemental distribution maps of Ti, Al, V, Zr, Co, Cu and Ni. The area marked by the dashed lines is β-Ti phase.

The EDS results show that the tiny phase in the yellow frame (Figure 2h) is a eutectoid microstructure composed of α-Ti, (Ti,Zr)_2_(Cu,Ni,Co) and β-Ti. In order to further observe the interface microstructure, the sample for TEM is prepared at the center of the yellow frame position shown in Figure 2h. Figure 4a shows the diffraction spots of each phase following calibration. Five kinds of contrast microstructures are found in the TEM images. Regions I and IV are calibrated as α-Ti, region II is β-Ti, and the bright white regions III and V are Ti_2_Cu. The contents of elements in each region are shown in Table 2.

Since Ti and Zr are the same main group elements, their chemical properties are similar and can be infinitely miscible. Cu, Ni and Co are near and infinitely miscible on the periodic table of elements. Therefore, the position of Ti in the Ti_2_Cu lattice can be replaced by Zr, and the position of Cu can be replaced by Ni and Co to form Ti_2_Ni, Zr_2_Cu, Zr_2_Ni, Ti_2_Co and Zr_2_Co IMCs, which can be expressed as (Ti,Zr)_2_(Cu,Ni,Co) IMCs in the joints with Zr-16Ti-6Cu-8Ni-6Co. Combined with the element content of the phase, regions III and V are (Ti,Zr)_2_(Cu,Ni,Co). Figure 4b shows that the Cu, Ni, Zr and Co elements are distributed in a similar area. The four elements are aggregated at the position of (Ti,Zr)_2_(Cu,Ni,Co), corresponding to the bright white area in the bright field image. Meanwhile, the region of poor Al corresponds to the β-Ti phase, which is consistent with the SAED results in Figure 4a.

**Figure 5:**
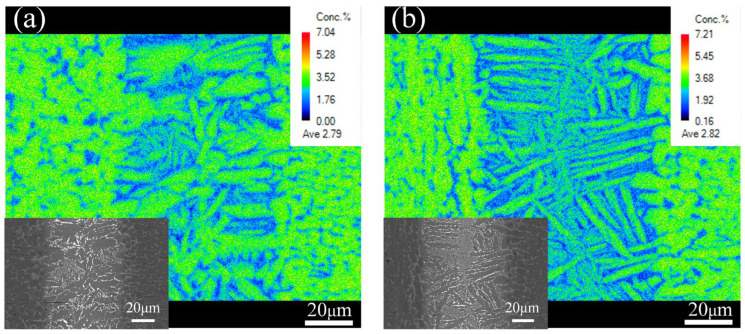
EPMA elemental mapping of Al in TC4 brazed joint with two kinds of brazing filler metals at 920 °C: (**a**) Zr-16Ti-9Cu-11Ni; (**b**) Zr-16Ti-6Cu-8Ni-6Co.

The β-Ti played a significant role in suppressing the (Ti,Zr)_2_(Cu,Ni,Co) IMCs in the central joints. According to Figure 4b, the β-Ti phase is low in Al phases. To compare the β-Ti contents of the joints brazed with Zr-16Ti-9Cu-11Ni and Zr-16Ti-6Cu-8Ni-6Co braze fillers, as shown in Figure 5, the EPMA result of the Al element is obtained. Since α-Ti is rich in Al, as shown in Table 1 Spot E, the large area of a yellow-green phase in the base metal is α-Ti, and the blue phase is β-Ti. It can be seen from the spectrum that the blue phase β-Ti in the brazed seam increases after the addition of the Co element. After calibration, the proportion of the blue phase β-Ti in the brazed seam increases from 34.95% to 56.26%. That explains the reason why the IMCs disappeared in the joints brazed at 900 °C after adding Co into the braze filler, while there were still residual IMCs in the joints when brazing without the Co addition.

### 3.2. Mechanical Properties of the TC4 Brazed Joint

**Figure 6:**
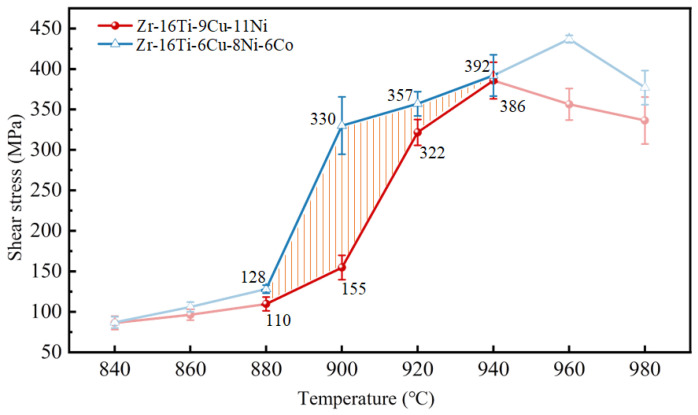
The shear strength of the joint with Zr-16Ti-9Cu-11Ni and Zr-16Ti-6Cu-8Ni-6Co at different brazing temperatures in weak erosion area (880–940 °C, marked by orange lines).

Figure 6 shows the results of the shear strength of the brazed joints under the different temperatures. According to the research of Zhang et al. [24], when brazing a TC4 alloy with a Zr-based filler alloy at temperatures higher than 950 °C, a strong erosion happened to the thin-walled TC4 alloy. So, the brazing temperature should be controlled at temperatures lower than 950 °C. Although some slight erosion happened when brazing the TC4 alloys at temperatures lower than 880 °C, the shear strength of the TC4 brazed joints was not desirable (lower than 150 MPa in this research) due to the residual IMC layer in the central joints. Therefore, we paid close attention to the brazing temperatures ranging from 880 to 950 °C, which can facilitate strong TC4 brazed joints with relatively weak erosion. By comparing the strength of the joints brazed at 880–950 °C, we can see that the shear strength of the Zr-16Ti-6Cu-8Ni-6Co brazed joint was always higher than that of the Zr-16Ti-9Cu-11Ni brazed joint at the same temperature. Specifically, when brazing at 900 °C, the shear strength of the joints brazed with Zr-16Ti-6Cu-8Ni-6Co filler metals was 330 MPa on average, which was almost 113% higher than that of the joints with Zr-16Ti-9Cu-11Ni filler metals. Meanwhile, the erosion of the Zr-16Ti-6Cu-8Ni-6Co brazed joint at 900 °C was weaker than that of the brazed joints at 920–980 °C. The enhancement of the joint strength might be due to the higher amounts of β-Ti phase formation in the TC4 brazed joints after adding Co into the Zr-based filler alloys. Since the Co element is defined as the slow eutectoid element of the Ti alloy [25], it will slow down the eutectoid reaction rate of the β-Ti phase. Under the general cooling rate, the eutectoid reaction cannot be carried out in time, leading to more residual β-Ti phases in the joints [16]. Moreover, β-Ti can dissolve more Cu and Ni elements than α-Ti, which resulted in suppressing the content of IMCs in the TC4 brazed joint. Nano-indentation tests were carried out on the α-Ti, β-Ti and (Ti,Zr)_2_(Cu,Ni,Co) phases, respectively. The results are shown in Table 3. It can be seen that the elastic modulus and hardness of β-Ti are lower than those of α-Ti, while (Ti,Zr)_2_(Cu,Ni,Co) has the highest elastic modulus and hardness. As a result, the β-Ti with a favorable plastic deformation ability can absorb more strain under the same stress state compared with α-Ti and (Ti,Zr)_2_(Ni,Cu,Co), which leads to the enhancement of the TC4 brazed joints.

**Figure 7:**
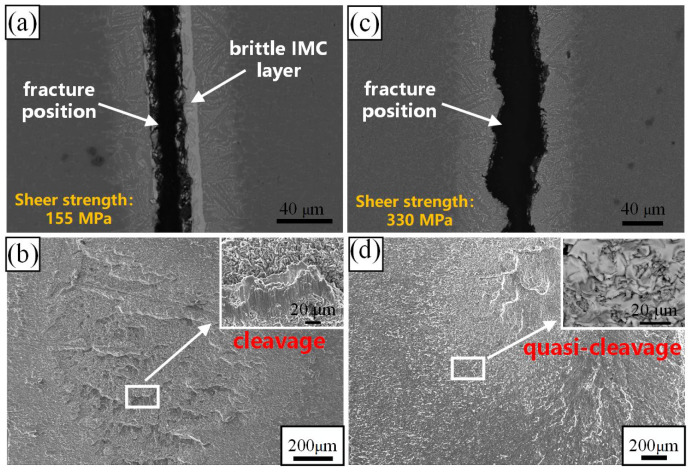
Fracture position and fracture surface of the brazed joints at 900 °C with (**a**), (**b**) Zr-16Ti-9Cu-11Ni and (**c**), (**d**) Zr-16Ti-6Cu-8Ni-6Co, respectively.

Figure 7 shows the fracture position and fracture surface of the brazed joints with Zr-16Ti-9Cu-11Ni and Zr-16Ti-6Cu-8Ni-6Co at 900 °C, respectively. The shear strength of the Zr-16Ti-9Cu-11Ni brazed joint with a brittle IMC layer is 155 MPa (Figure 6), and the Zr-16Ti-6Cu-8Ni-6Co brazed joint without a brittle IMC layer can achieve 330 MPa. It can be seen from the test results that the existence of a brittle IMC layer is the decisive factor for the shear strength of the brazed joint. As shown in Figure 7b, it can be seen that the fracture morphology of the Zr-16Ti-9Cu-11Ni brazed joint has an obvious cleavage surface, which is a typical feature of a brittle fracture. The fracture surface of the Zr-16Ti-6Cu-8Ni-6Co brazed joint is dominated by a quasi-cleavage (Figure 7d).

## 4. Conclusions

In this study, the microstructure and properties of brazed joints with Zr-16Ti-9Cu-11Ni and Zr-16Ti-6Cu-8Ni-6Co fillers were compared. The microstructure analysis and comparison were carried out at different temperatures. The specific conclusions are as follows:

(1) The Zr-16Ti-6Cu-8Ni-6Co amorphous filler was designed and prepared by adding a slow eutectoid Co element. After the addition of Co to the Zr-16Ti-9Cu-11Ni filler, the brazing temperature for eliminating the brittle IMC layer was reduced from 920 °C to 900 °C. The β-Ti phase dissolved more Cu and Ni elements than the α-Ti. The brittle IMC layer in the Zr-16Ti-6Cu-8Ni-6Co joint completely disappeared and was replaced by a uniform Widmanstatten microstructure at 900 °C.
(2) The TEM result shows that the phase in the brazed seam of Zr-16Ti-6Cu-8Ni-6Co brazed joint is a eutectoid microstructure composed of α-Ti, (Ti,Zr)_2_(Cu,Ni,Co) and β-Ti. The (Ti,Zr)_2_(Cu,Ni,Co) is composed of Ti_2_Ni, Zr_2_Cu, Zr_2_Ni, Ti_2_Co and Zr_2_Co IMCs. Meanwhile, the region of poor Al corresponds to the β-Ti phase.
(3) The strength of the Zr-16Ti-6Cu-8Ni-6Co brazed joint is higher than that of the Zr-16Ti-9Cu-11Ni brazed joint at a weak erosion degree (<950 °C). The shear strength of the Zr-16Ti-6Cu-8Ni-6Co brazed joint increased by 113% when the brittle IMC layer was eliminated (at 900 °C).
(4) The addition of Co to the Zr-based filler increases the content of β-Ti by 21.31% compared with the brazed joint with Zr-16Ti-9Cu-11Ni. The elastic modulus of β-Ti (143 GPa) is lower than that of α-Ti (169 GPa) and (Ti,Zr)_2_(Ni,Cu,Co) (203 GPa), and the β-Ti is subjected to a greater strain under the same stress state. Under the same brazing temperature, the strength of the Zr-16Ti-6Cu-8Ni-6Co brazed joint is higher than that of the Zr-16Ti-9Cu-11Ni brazed joint.

## Figures and Tables

**Table 1:** The element content of each phase of Zr-16Ti-6Cu-8Ni-6Co brazed joint (at.%).

	Ti	Zr	Al	V	Cu	Ni	Co	Possible Phase
A	33.26	59.60	0.50	0.29	1.89	2.74	1.72	(Ti,Zr)_(s.s)_
B	31.87	32.40	3.06	0.82	8.53	13.72	9.60	(Ti,Zr)_2_(Cu,Ni,Co)
C	80.71	6.38	6.73	3.92	0.73	0.48	1.05	α-Ti
D	64.47	14.55	9.17	2.74	2.17	4.15	2.75	β-Ti
E	80.33	7.58	10.57	0.67	0.45	0.22	0.18	α-Ti
F	59.71	16.21	8.71	2.69	3.51	5.93	3.23	β-Ti

**Table 2:** The element content of FIB sample in Figure 2h (at.%).

	Ti	Zr	Al	V	Cu	Ni	Co	Possible Phase
I	33.26	59.60	0.50	0.29	1.89	2.74	1.72	α-Ti
II	31.87	32.40	3.06	0.82	8.53	13.72	9.60	β-Ti
III	80.71	6.38	6.73	3.92	0.73	0.48	1.05	(Ti,Zr)_2_(Cu,Ni,Co)
IV	64.47	14.55	9.17	2.74	2.17	4.15	2.75	α-Ti
V	80.33	7.58	10.57	0.67	0.45	0.22	0.18	(Ti,Zr)_2_(Cu,Ni,Co)

**Table 3:** The elastic modulus and hardness of each microstructure of the Zr-16Ti-6Cu-8Ni-6Co brazed joint at 840 °C.

Phase	Elastic Modulus (GPa)	Hardness (GPa)
α-Ti	169	6.379
β-Ti	143	5.021
(Ti,Zr)_2_(Ni,Cu,Co)	203	7.586

## Data Availability

The original contributions presented in this study are included in the article, and further inquiries can be directed to the corresponding author.

## References

[1] Zhang Z.H., Liu Q.M., Yang H.Y., Liu S.F. (2017). Room-Temperature Compressive Deformation Behavior of High-Strength Ti-15V-3Al-3Cr-3Sn-1Nb-1Zr Alloy. J. Mater. Eng. Perform..

[2] Li R.G., Qin T., Fei A.G., Wang H.M., Zhao Y.T., Chen G., Kai X.Z. (2019). Performance and Microstructure of TC4 Titanium Alloy Subjected to Deep Cryogenic Treatment and Magnetic Field. J. Mater. Sci. Chem. Phys..

[3] Jing Y.J., Yang H.B., Shang Y.L., Xiong H.P. (2021). The Design of a New Ti-Zr-Cu-Ni-Ag Brazing Filler Metal for Brazing of Titanium Alloys. Weld. World.

[4] Williams J.C., Boyer R.R. (2020). Opportunities and Issues in the Application of Titanium Alloys for Aerospace Components. Metals.

[5] Yang M., Li S., Zhang X.J., Yang H.L., Nie L.P., Wu X. (2022). Effect of Brazing Temperatures on Microstructure and Properties of TC4/Ti_57_Zr_13_Cu_21_Ni_9_/316L. Met. Res. Technol..

[6] Liang M., Qin Y., Zhang D., Zhao F. (2022). Microstructural Evolution and Mechanical Properties of Vacuum Brazed TC4 Titanium Alloy Joints with Ti-Zr-Ni Filler Metal. J. Mater. Eng. Perform..

[7] Zhang X.P., Shi Y.W., Ren Y.W. (1995). Study on the Dissolution Model of Base Metal in the Liquid Nickel-Based Amorphous and Crystalline Brazing Filler Metals during Vacuum Brazing. J. Aeronaut. Mater..

[8] Zhang X.P., Shi Y.W., Ren Y.W. (1996). Study on the Dissolution Characteristics of Steel Base Metal in the Liquid Nickel-Based Amorphous and Crystalline Brazing Filler Metals during Vacuum Brazing Process. J. Aeronaut. Mater..

[9] Li Y., Liu W., He P., Feng J., Sekulic D.P. (2013). Dissolution of TiAl Alloy during High Temperature Brazing. J. Mater. Sci..

[10] Takemoto T., Okamoto I. (1988). Intermetallic Compounds Formed During Brazing of Titanium with Aluminum Filler Metals. J. Mater. Sci..

[11] Pang S.J., Sun L.L., Xiong H.P., Chen C., Liu Y., Li H.F., Zhang T. (2016). A Multicomponent TiZr-Based Amorphous Brazing Filler Metal for High-Strength Joining of Titanium Alloy. Scr. Mater..

[12] Liu S. (2020). Microstructure and Filler Metal Element Diffusion Behavior of TiZrCuNi Filler Metal Vacuum Brazing Pure Titanium TA1 Joint Interface. Mater. Mech. Eng..

[13] Bai X., Liu M., Pang W., Xiong H., Ren X., Ren H., Zhang T. (2023). Novel Ti–Zr–Co–Cu–M (M = Sn, V, Al) Amorphous/Nanocrystalline Brazing Fillers for Joining Ti–6Al–4V Alloy. Mater. Charact..

[14] Wang Y., Jiao M., Yang Z.W., Wang D.P., Liu Y.C. (2018). Vacuum Brazing of Ti_2_AlNb and TC4 Alloys Using Ti-Zr-Cu-Ni and Ti-Zr-Cu-Ni plus Mo Filler Metals: Microstructural Evolution and Mechanical Properties. Arch. Civ. Mech. Eng..

[15] Ma T.J., Kang H., Qu P. (2005). Effects of the Brazing Properties of TC4 Alloy by Addition of Mixed Rare Earth on the Ti Base Brazing Filler Metals. Aviat. Precis. Manuf. Technol..

[16] He Y.M., Lu C.Y., Ni C.Y., Chen Q.X., Zheng W.J., Wang D.H., Wei L.F., Wang L.M., Sun Y., Zou H. (2020). Tailoring Microstructure and Mechanical Performance of the TC4 Titanium Alloy Brazed Joint through Doping Rare-Earth Element Dy into Ti-Cu-Ni Filler Alloy. J. Manuf. Process..

[17] Zhong S.J., Liu P., Qin J., Si H., Long W.M., Fang N.W. (2022). Research Progress of Brazing Titanium Alloy Plate Fin Heat Exchanger. Electr. Weld. Mach..

[18] Wang J., Li Y.J. (2016). Brazing and Diffusion Welding Technology.

[19] Peker A., Johnson W.I. (1993). A Highly Processable Metallic-Glass: Zr41.2ti13.8cu12.5ni10.0be22.5. Appl. Phys. Lett..

[20] Ganjeh E., Sarkhosh H. (2013). Microstructural, Mechanical and Fractographical Study of Titanium-CP and Ti-6Al-4V Similar Brazing with Ti-Based Filler. Mater. Sci. Eng. Struct. Mater. Prop. Microstruct. Process..

[21] Lee M.K., Lee J.G. (2013). Mechanical and Corrosion Properties of Ti–6Al–4V Alloy Joints Brazed with a Low-Melting-Point 62.7Zr–11.0Ti–13.2Cu–9.8Ni–3.3Be Amorphous Filler Metal. Mater. Charact..

[22] Han D., Zhao Y., Zeng W. (2021). Effect of Zr Addition on the Mechanical Properties and Superplasticity of a Forged SP700 Titanium Alloy. Materials.

[23] Yuan B.G., Li C.F., Yu H.P., Sun D.L. (2010). Influence of Hydrogen Content on Tensile and Compressive Properties of Ti-6Al-4V Alloy at Room Temperature. Mater. Sci. Eng. Struct. Mater. Prop. Microstruct. Process..

[24] Zhang L.X., Ding Y.H., Sun M.J., Zhang B.Y., Sun Z., Chang Q., Zhang B., Long W.M. (2024). The Microstructure and Properties of TC4 Brazed with a Newly Designed Low-Erosion Zr-Based Brazing Filler. Mod. Transp. Met. Mater..

[25] Han D., Zhao Y., Zeng W., Xiang J. (2021). Investigation of Slow Eutectoid Element on Tensile Properties and Superplasticity of a Forged SP700 Titanium Alloy. Metals.

